# Characteristics, Management, and Outcomes of Patients With Osteosarcoma: An Analysis of Outcomes From the National Cancer Database

**DOI:** 10.5435/JAAOSGlobal-D-22-00009

**Published:** 2022-02-22

**Authors:** Taylor D. Ottesen, Blake N. Shultz, Alana M. Munger, Cosmas Sibindi, Alp Yurter, Arya G. Varthi, Jonathan N. Grauer

**Affiliations:** From the Department of Orthopaedics and Rehabilitation, Yale School of Medicine, New Haven, CT (Dr. Ottesen, Shultz, Dr. Munger, Sibindi, Yurter, Dr. Varthi, and Dr. Grauer), and the Harvard Combined Orthopedic Residency Program, Harvard Medical School, Boston, MA (Dr. Ottesen).

## Abstract

**Introduction:**

Previous studies about osteosarcoma patient characteristics, management, and outcomes have limited patient numbers, combine varied tumor types, and/or are older studies.

**Methods:**

Patients with osteosarcoma from the 2004 to 2015 National Cancer Database data sets were separated into axial, appendicular, and other. Demographic and treatment data as well as 1-, 5-, and 10-year survival were determined for each group. A multivariate Cox analysis of patient variables with the likelihood of death was performed, and the Kaplan Meier survival curves were generated.

**Results:**

Four thousand four hundred thirty patients with osteosarcoma (3,435 appendicular, 810 axial, and 185 other) showed survival at 1-year, 5-year, and 10-year and was highest among the appendicular cohort (91.17%, 64.43%, and 58.58%, respectively). No change in survival was seen over the periods studied. The likelihood of death was greater with increasing age category, distant metastases, and treatment with radiation alone but less with appendicular primary site, treatment with surgery alone, or surgery plus chemotherapy.

**Discussion:**

Despite advances in tumor management, surgical excision remains the best predictor of survival for osteosarcomas. No difference was observed in patient survival from 2004 to 2015 and, as would be expected, distant metastases were a poor prognostic sign, as was increasing age, male sex, and axial location.

Osteosarcoma is the most common primary osseous sarcoma.^1,2^ The incidence has been estimated to be approximately 5.0 per million patients aged zero to 19^1^ and 3.5 per million patients for patients aged older than 60.^3^ Osteosarcoma has a predilection for invading the metaphysis of the long bones of the appendicular skeleton, chiefly the distal femur and proximal tibia.^4^ Osteosarcoma can also affect the axial skeleton/other locations, although this is less common.^4^

Owing to the relatively rare nature of osteosarcoma, previous studies have been limited in sample size (ranging from 34 to 648).^5,6,7,8,9^ These have tended to show positive prognostic factors to include but are not limited to younger age at the time of diagnosis,^1^ lack of metastases at the time of diagnosis,^5^ time to treatment initiation,^10^ parosteal subtype, smaller tumor size, and greater than 90% necrosis with chemotherapy.^11^ Depending on the combination of these prognostic factors, 5-year survival rates for osteosarcoma have been reported to vary from 70% in patients with local disease at the time of diagnosis to 20% in patients with metastatic or recurrent disease.^12^

The mainstay of treatment for osteosarcoma consists of surgical resection and reconstruction along with neoadjuvant chemotherapy (before surgery) and adjuvant chemotherapy (after surgery). Despite advances and numerous attempts to refine treatment regimens, the survival rates of patients with osteosarcoma have largely remained unchanged over the last 3 decades.^13^ Radiation therapy is not a mainstay in the treatment of osteosarcoma and is largely reserved for cases without negative resection margins.^14,15^ No treatment is rarely considered except in cases of exceedingly poor prognosis/patient choice.

Previous studies investigating treatment management of osteosarcoma have largely been limited by patient numbers. The National Cancer Database (NCDB) contains patient information from multiple centers across the United States with more than 30 million patient records representing a unique opportunity to learn additional insights concerning this patient population.^16,17^ To our knowledge, no previous studies assessing the outcomes in patients with primary osteosarcoma using the NCDB database exist. Thus, this study sought to evaluate the incidence, treatment, and outcomes of patients with osteosarcoma using the large, robust, and multicenter national patient population found within the NCDB database to further characterize the osteosarcoma population, treatments, and survivals.

## Methods

### Data Source and Study Population

The NCDB was used to conduct a retrospective database cohort study. This database represents an initiative of the American College of Surgeons and American Cancer Society to track and improve outcomes in cancer.^17^ The data contain information gathered from multiple centers, with demographic variables and outcome variables on more than 70% of new cancer diagnoses in the United States.^16-18^ Data are collected until either death of the patient or the patient is lost to follow-up.

Patients diagnosed with osteosarcoma were identified from the 2004 to 2015 NCDB databases by International Classification of Diseases for Oncology, third edition histology codes 9180, 9183, 9185, 9187, 9192, 9193, and 9194. Osteosarcoma cases were then divided into three cohorts by the site of the primary tumor: axial (International Classification of Diseases for Oncology, third edition codes C41.0, C41.1, C41.2, and C41.4), appendicular (C40.0, C40.1, C40.2, C40.3, and C40.9), or other location (C40.8, C41.3, C41.8, and C41.9). Patients were excluded if treatment was performed at another location than the reporting facility (Class of Case = 00), along with those missing data related to the presence of metastases at diagnosis.

### Population Characteristics

Demographic variables extracted from the database include age, sex, Charles-Deyo score, and metastasis at presentation. Patients were also split into 3 era groups based on year of diagnosis: 2004 to 2007, 2008 to 2011, and 2012 to 2015.

Treatment data including surgery, radiation, chemotherapy, and combination treatments were also extracted from the database. Finally, 1-, 5-, and 10-year survival was calculated for each primary site cohort.

### Comparison of Populations

Bivariate analyses were used to compare preoperative and surgical variables between the axial, appendicular, and other cohorts. Pearson chi-squared test was used for categorical variables, one-way Analysis of Variance (ANOVA) for continuous variables, and the Kruskal-Wallis test for ordinal variables (American Society of Anesthesiologists (ASA) class).

To study the influence of preoperative variables and treatment choice on the likelihood of death, a multivariate Cox analysis was conducted for the axial and appendicular cohorts. Incidence rate ratio (IRR) was calculated, which is a more accurate measure of the relative effect of a given exposure on the risk of the occurrence of an event between the exposed and unexposed populations. It takes the incidence rate (defined as the number of events per person time–person-years in this study) of the exposed population and divides this by all the people at risk for an outcome (in this instance death) at any one point in time. IRR is useful when calculating relative risk over a period when the population at risk is constantly changing (because of deaths or remission).^19^

Finally, the Kaplan Meier survival curves were generated showing long-term survival of various cohorts. First, long-term survival was shown for patients with and without distant metastases at presentation. Then, long-term survival was shown for the appendicular, axial, and other cohorts. Finally, long-term survival was shown for each of the 3 era groups.

All statistical analyses were conducted using Stata version 13.0 (StataCorp, LP). Significance was set at *P* < 0.05.

## Results

### Population and Treatment Characteristics

Of the 4,430 patients identified with osteosarcoma, 3,435 were classified as appendicular (77.54%), 810 cases were classified as axial (18.28%), and 185 were other (4.18%). These cohorts are defined in Table 1. The appendicular group was younger and healthier (lower Charles-Deyo score) than the axial and other groups. Metastases were present for a similar percent of cases for the appendicular (18.05%) and axial (18.40%) groups (but lower than the other group, 40.0%). No statistically significant difference existed is the distribution between the appendicular, axial, and other between the years of diagnosis.

**Table 1:** Comparison of Demographics Between the Appendicular, Axial, and Other Osteosarcoma Cohorts

Patient Characteristic	Total	Appendicular	Axial	Other	*P*
No. of patients	4,430	3,435	810	185	
Mean age in yrs (±SD)	29.53 ± 20.32	25.52 ± 17.99	43.08 ± 21.11	44.57 ± 24.51	**<0.001**
Women	1,963 (44.31)	1,495 (43.52)	383 (47.28)	85 (45.95)	0.138
Charles-Deyo score					**<0.001**
0	4,021 (90.77)	3,157 (91.91)	699 (86.30)	165 (89.19)	
1	348 (7.86)	242 (7.05)	92 (11.36)	14 (7.57)	
2	50 (1.13)	28 (0.82)	16 (1.98)	6 (3.24)	
3	11 (0.25)	8 (0.23)	3 (0.37)	0 (0.00)	
Metastasis at presentation					**< 0.001**
No	3,587 (80.97)	2,815 (81.95)	661 (81.60)	111 (60.00)	
Yes	843 (19.03)	620 (18.05)	149 (18.40)	74 (40.00)	
Year of diagnosis^a^					0.167
2004-2007	1,574 (35.53)	1,233 (35.90)	288 (35.56)	53 (28.65)	
2008-2011	1,607 (36.28)	1,227 (35.72)	298 (36.79)	82 (44.32)	
2012-2015	1,249 (28.19)	975 (28.38)	224 (27.65)	50 (27.03)	

a No statistically significant difference exists between age, sex, Charles-Deyo score, and the prevalence of distant metastases at presentation between the 3 era groups in the appendicular or axial cohorts.

Significance set at <0.05.

Treatment data for the axial, appendicular, and other cohorts are shown in Table 2. The appendicular group was more likely to receive surgery and radiation but less likely to receive isolated radiation than the axial or other groups. For both the axial and other cohorts, radiation therapy was more likely to be given postoperatively (axial: 13.58%; other: 9.73%) than preoperatively (axial: 1.60%; other: 2.16%) or intraoperatively (axial: 0.12%; other: 0.00%). Regarding chemotherapy, the appendicular cohort was more likely to receive chemotherapy (83.00%) than the axial (64.81%) and other (67.03%) cohorts. In treatment combinations, surgery and chemotherapy were the most common for all scenarios but more common in appendicular cases (69.43%) than axial cases (34.57%) or other cases (41.08%).

**Table 2:** Comparison of Surgical Data and Long-Term Survival for the Appendicular and Axial Cohorts

Patient Characteristic	Total	Appendicular	Axial	Other	*P*
No. of patients	4,430	3,435	810	185	
Surgical treatment					**<0.001**
No surgery	755 (17.04)	470 (13.68)	225 (27.78)	60 (32.43)	
Surgery	3,669 (82.82)	2,959 (86.14)	585 (72.22)	125 (67.57)	
Unknown	6 (0.14)	6 (0.17)	0 (0.00)	0 (0.00)	
Radiation therapy					**<0.001**
None	4,147 (93.61)	3,321 (96.69)	667 (82.35)	159 (85.95)	
Preoperative	31 (0.70)	14 (0.41)	13 (1.60)	4 (2.16)	
Postoperative	182 (4.11)	454 (1.57)	110 (13.58)	18 (9.73)	
Both or intraoperative	8 (0.18)	4 (0.12)	4 (0.49)	0 (0.00)	
Unknown	62 (1.40)	42 (1.22)	16 (1.98)	4 (2.16)	
Chemotherapy					**<0.001**
No (0)	862 (19.46)	542 (15.78)	261 (32.22)	59 (31.89)	
Yes (1)	3,500 (79.01)	2,851 (83.00)	525 (64.81)	124 (67.03)	
Unknown (2)	68 (1.53)	42 (1.22)	24 (2.96)	2 (1.08)	
Treatment combination					**<0.001**
No treatment	274 (6.19)	152 (4.43)	96 (11.85)	26 (14.05)	
Isolated surgery	625 (14.11)	445 (12.95)	157 (19.38)	23 (12.43)	
Isolated radiation	6 (0.14)	1 (0.03)	3 (0.37)	2 (1.08)	
Isolated chemotherapy	570 (12.87)	381 (11.09)	152 (18.77)	37 (20.00)	
Surgery and radiation	69 (1.56)	16 (0.47)	40 (4.94)	13 (7.03)	
Surgery and chemotherapy	2,741 (61.87)	2,385 (69.43)	280 (34.57)	76 (41.08)	
Chemotherapy and radiation	2 (0.05)	1 (0.03)	1 (0.12)	0 (0.00)	
Surgery, radiation, and chemotherapy	143 (3.23)	54 (1.57)	81 (10.00)	8 (4.32)	
Survival					**<0.001**
1-yr survival (%)	87.1	91.17	74.22	67.33	
5-yr survival (%)	60.17	64.43	45.97	42.57	
10-yr survival (%)	53.92	58.58	38.27	NA	

No statistically significant difference exists between the era groups for the above variables, within the appendicular or axial cohorts.

Significance set at <0.05.

### Survival Data and Kaplan Meier Curves

Survival is also shown in Table 2. The appendicular cohort demonstrated a statistically higher 1-year survival rate (91.17%), 5-year survival rate (64.43%), and 10-year survival rate (58.58%) than the other two cohorts (axial: 1-year [74.22%], 5-year [45.97%], and 10-year [38.27%]; other: 1-year [67.33%], 5-year [45.57%], and 10-year [N/A]).

**Figure 1:**
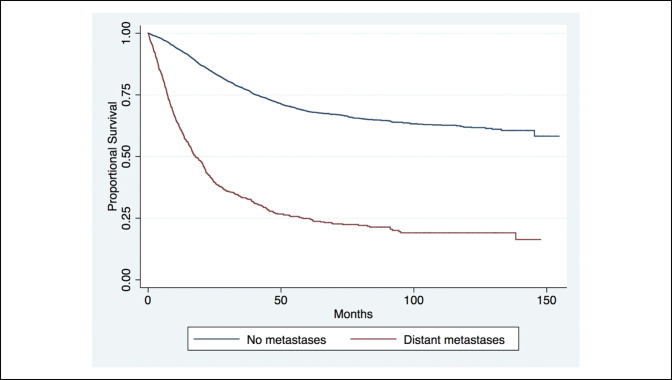
Chart showing the long-term survival of all patients with and without distant metastases at the time of presentation (*P* < 0.001).

**Figure 2:**
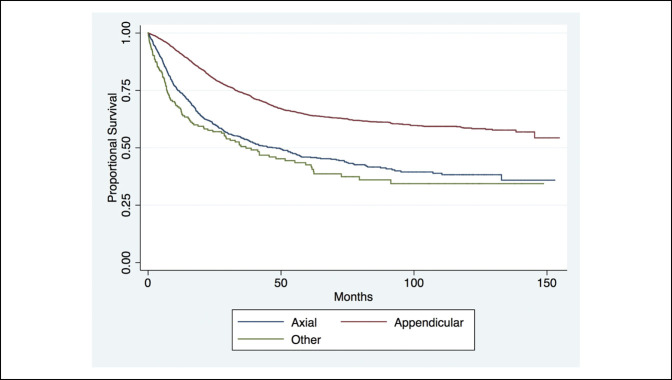
Chart showing the long-term survival of patients in the axial, appendicular, and other cohorts (*P* < 0.001).

**Figure 3:**
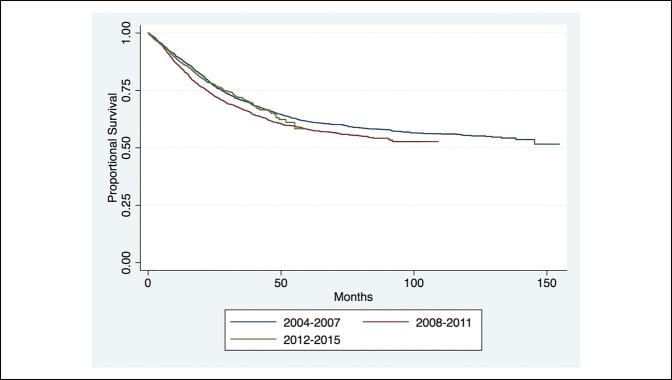
Chart showing the long-term survival of patients in the 3 era groups (*P* = 0.220)

The Kaplan Meier curve analysis of proportional survival of patients with distant metastases on diagnosis revealed markedly decreased life expectancy in patients with distant metastases at 1, 5, and 10 years from time of diagnosis (Figure 1). The long-term survival of axial, appendicular, and other cohorts showed markedly a poorer long-term survival at 1, 5, and 10 years of those with tumors in the axial or other cohorts (Figure 2). Furthermore, no notable difference was observed in the long-term survival for patients diagnosed since the mid-2000s (Figure 3).

### Multivariate Analyses

Looking at axial and appendicular cohorts individually, Table 3 demonstrates the multivariate Cox analysis of the likelihood of death at any given time for demographic and surgical variables for just the axial cohort. The likelihood of death increased in a sequential manner with increasing age category (23 to 45 age cohort: IRR = 1.325, *P* < 0.001; 46 to 62 age cohort: IRR = 2.107, *P* < 0.001; 62+ age cohort: IRR = 3.528, *P* < 0.001) and the presence of metastasis at the time of diagnosis demonstrated (IRR = 3.958, *P* < 0.001). The likelihood of death decreased with female sex (IRR = 0.798, *P* < 0.001). No significant change existed in the IRR with the year of diagnosis. The likelihood of death demonstrated an increase with the treatment choice of radiation alone (IRR = 16.575, *P* = 0.006), whereas the likelihood of death demonstrated a decrease with the treatment choice was surgery alone (IRR = 0.305, *P* < 0.001) and surgery with chemotherapy (IRR = 0.476, *P* < 0.001).

**Table 3:** A Multivariate Cox Analysis of the Likelihood of Death at Any Given Time for Demographic and Surgical Variables for the Axial Cohort

Likelihood of Death at Any Given Time	IRR^a^	95% Confidence Interval			*P*
Age category					
<23	Ref	Ref		Ref	Ref
23-45	1.325	1.137	—	1.545	**<0.001**
46-62	2.107	1.734	—	2.561	**<0.001**
62+	3.528	2.695	—	4.619	**<0.001**
Sex					
Men	Ref	Ref		Ref	Ref
Women	0.798	0.707	—	0.9	**<0.001**
Metastasis at time of diagnosis					
Yes	3.958	3.478	—	4.505	**<0.001**
Year of diagnosis					
2004-2007	Ref	Ref		Ref	Ref
2008-2011	1.102	0.966	—	1.258	0.149
2012-2015	0.965	0.815	—	1.142	0.677
Treatment choice					
None	Ref	Ref		Ref	Ref
Surgery	0.305	0.227	—	0.411	**<0.001**
Radiation	16.575	2.263	—	121.416	**0.006**
Chemotherapy	0.919	0.707	—	1.193	0.525
Surgery and radiation	0.599	0.309	—	1.165	0.131
Surgery and chemotherapy	0.476	0.375	—	0.605	**<0.001**
Radiation and chemotherapy	5.62	0.767	—	41.161	0.089
Unknown	0.363	0.217	—	0.606	**<0.001**

IRR = Incidence Rate Ratio

a Defined as the incidence rate of the exposed population divided by the total number of people at risk of death at any one point in time.

Significance set at <0.05.

Table 4 demonstrates the multivariate Cox analysis of the likelihood of death at any given time for demographic and surgical variables for the appendicular cohort. In similarity to the axial cohort, the likelihood of death increased in a sequential manner with increasing age category (23 to 45 age cohort: IRR = 1.496, *P* = 0.012; 46 to 62 age cohort: IRR = 2.243, *P* < 0.001; 62+ age cohort: IRR = 4.088, *P* < 0.001) and the presence of metastasis at time of diagnosis (IRR = 3.393, *P* < 0.001). Unlike the axial cohort, no statistically significant change existed in the IRR with female sex. No statistically significant change existed in the IRR with the year of diagnosis. The likelihood of death demonstrated a statistically significant increase with the treatment choice of chemotherapy alone (IRR = 1.682, *P* = 0.001), whereas the likelihood of death demonstrated a statistically significant decrease with the treatment choice was surgery alone (IRR = 0.443, *P* < 0.001).

**Table 4:** A Multivariate Cox Analysis of the Likelihood of Death at Any Given Time for Demographic and Surgical Variables for the Appendicular Cohort

Likelihood of Death at Any Given Time	IRR^a^	95% Confidence Interval			*P*
Age category					
<23	Ref	Ref		Ref	Ref
23-45	1.496	1.094	—	2.045	**0.012**
46-62	2.243	1.614	—	3.117	**<0.001**
62+	4.088	2.776	—	6.020	**<0.001**
Sex					
Men	Ref	Ref		Ref	Ref
Women	0.782	0.641	—	0.955	0.016
Metastasis at time of diagnosis					
Yes	3.393	2.659	—	4.330	**<0.001**
Year of diagnosis					
2004-2007	Ref	Ref		Ref	Ref
2008-2011	1.008	0.804	—	1.263	0.947
2012-2015	0.844	0.644	—	1.106	0.218
Treatment choice					
None	Ref	Ref		Ref	Ref
Surgery	0.443	0.309	—	0.637	**<0.001**
Radiation	4.533	1.044	—	19.683	0.044
Chemotherapy	1.682	1.241	—	2.279	**0.001**
Surgery and radiation	0.840	0.514	—	1.372	0.486
Surgery and chemotherapy	0.788	0.592	—	1.049	0.103
Radiation and chemotherapy	8.244	1.046	—	64.997	0.045
Unknown	0.883	0.533	—	1.465	0.631

IRR = Incidence Rate Ratio

a Defined as the incidence rate of the exposed population divided by the total number of people at risk of death at any one point in time.

Significance set at <0.05.

## Discussion

Osteosarcoma remains a clinical challenge. This study evaluated 4,430 patients with osteosarcoma from the NCDB database, which is the largest osteosarcoma cohort presented to date in the literature. Despite advances in tumor management, long-term survival of patients with osteosarcoma was found to be similar over the last 15 years. As expected, metastasis at presentation heralded a worse prognosis and being a surgical candidate was correlated with increased life-expectancy.

Most cases identified were appendicular in nature. This is consistent with known epidemiology of osteosarcoma.^20^ In fact, 77.5% of the cases were appendicular. The second most common site was axial (18.3%). The least common site was other (eg, head, neck, or mandible, 4.2%). Again, these figures are consistent with previous studies and substantiate the representative nature of the National Trauma Data Bank (NTDB) population.

The overall 1-year osteosarcoma survival was 87.1%, the 5-year survival was 60.17%, and then 10-year survival was 53.92%, which is consistent with previous reports.^3,21^ These figures were slightly better for the appendicular cohort (1-year 91.17%, 5-year 64.43%, and 10-year 58.58%), but note that the overall numbers were largely driven by the appendicular numbers because of their overwhelming majority of the study population. Conversely, there was worse survival for the axial cohort (1-year 74.22%, 5-year 45.97%, 10-year 38.27%) presumably because of later stage diagnosis and more limited surgical options. The other group was more similar to axial group for presumed similar reasons.

Surgical excision of osteosarcoma is widely held as the best treatment option when possible. It is thus not surprising that when surgery is performed, outcomes are the highest. Surgical intervention addresses the source of the neoplasm. When examining the progress in overall survival over the past few decades, the increase in survival in the 1970s was attributed to the addition of chemotherapy to osteosarcoma treatment regimens.^22^ Although there have been advances in chemotherapy, a prospective study examined 31 consecutive patients with local osteosarcomas who were treated exclusively with chemotherapy found that only 3 of the 31 patients were cured exclusively with chemotherapy, suggesting that chemotherapy alone is insufficient for the management of osteosarcoma.^23^

It has also been shown that there has not been an increase in 5-year overall survival from the 1980s to the 2000s.^22^ The current investigation was able to evaluate for possible more recent changes in survival (time intervals evaluated were 2004 to 2007, 2008 to 2011, and 2012 to 2015). This study also found no increases were found in survival noted over these time intervals. Certainly, new treatment paradigms exist for osteosarcoma, including immune-based therapies—including inhaled aerosolized granulocyte-macrophage colony-stimulating factor and liposomal muramyl tripeptide phosphatidylethanolamine—to treat and prevent lung metastasis as well as using antitumor agents and antiangiogenesis agents^24^; however, the impact of such interventions may not be fully reflected in the time frame studied because of a lack of 10-year follow-up data for more recent years in the data set.

This investigation also demonstrated that axial disease and metastasis at the initial presentation were poor predictors of long-term survival, which is consistent with previous investigations.^25,26^ One may postulate that there is a multitude of surgical interventions to resect a tumor within the appendicular skeleton,^27^ whereas a tumor that originates in the axial skeleton may prove difficult and more complex for complete en bloc resection. Regarding the importance of primary metastasis, Ozaki et al^25^ examined the overall survival of 67 patients with high-grade osteosarcoma of the pelvis and found that primary metastasis represented a statistically significant poor prognostic factor. One may postulate that osteosarcomas with metastatic potential may represent a more advanced or aggressive lesion in comparison to those without metastasis, thereby conferring decreased overall survival.

Increased age was associated with worse survival. It should be noted that this may represent a confounding variable; however, an analysis of the demographic data between the cohorts demonstrated that a statistically significant difference existed in the age between the cohorts, with the appendicular cohort demonstrating a younger age in comparison to the axial cohort. One may propose that the decrease in the age of osteosarcoma within the appendicular skeleton is because the appendicular skeletal has the most areas with high epiphyseal plate proliferation,^28^ which would inevitably decrease the age range for the appendicular cohort. A previous investigation has demonstrated that patients older the age of 65 demonstrated worse prognosis because of more metastatic disease at initial presentation, fewer patients receiving chemotherapy, and more patients excluded from clinical trials.^29^ In a retrospective review of 438 patients with osteosarcoma, however, age was not a notable independent prognostic indicator of the overall survival or disease-free survival.^30^ Although age may represent a confounding variable, previous research has shown that tumor site and primary metastasis were statistically notable poor prognostic factors on a multivariate Cox regression analysis, which would have taken age into consideration.^26^

The fact that male sex represents a poor prognostic factor is in concordance with previous investigations that have demonstrated that male sex is associated with delayed presentation and worse outcomes for the same level of disease in comparison to the female sex.^31,32^ Bieleck et al^31^ examined 1,702 consecutive newly diagnosed patients with high-grade osteosarcoma and found that male sex was associated with poor response to chemotherapy on both univariate and multivariate analysis. However, it should be noted that on univariate analysis, male sex was not as statistically significant in comparison to tumor site, duration of symptoms before presentation, and treatment delay. In addition, male sex was not significantly associated with poor overall survival or event-free survival.^31^

There have also been advances regarding the surgical technique in performing surgical resections of osteosarcomas. Computer-assisted tumor surgery, using three-dimensional image-guided bone resection, may increase the accuracy of complex tumor resections,^33-35^ although there are issues with computer-assisted tumor surgery, including a learning curve to become comfortable with the technology and a mismatch between preoperative images and actual patient anatomy.^33^ More extensive research is needed to investigate whether technology-assisted tumor surgery helps to improve the overall survival and disease-free survival in patients with osteosarcoma.

Limitations exist to this study. The data used in this study were acquired from the NCDB and therefore may demonstrate an overrepresentation of academic institutions with access to clinical trials. However, given the fact that the NCDB represents more than 70% of new cancer diagnoses in the United States and contains more than 30 million patient records,^16,17^ it is believed that this is a patient cohort that could be generalizable. Given the fact that the data within the NCDB are gathered from numerous institutions, the collection of these data may in fact be heterogenous. However, as multiple of the variables examined where objective in nature, it is believed this would not markedly affect the results of this investigation. Finally, tumor and treatment specifics (such as radiation-induced osteosarcomas) beyond general categories may affect outcomes and/or classifications and are not captured by the data set but may have an impact on survival.

## Conclusion

In conclusion, this study evaluated more than 4,000 patients with osteosarcoma and defined modern survival rates as largely unchanged from more dated studies. Surgical excision remains the best predictor of survival for osteosarcomas. Furthermore, factors correlating with decreased survival include distant metastases, increasing age, male sex, and axial location. With survival having remained similar over the time frames studied, the need for further advances in this area are highlighted and leaves room for additional studies as cancer care progresses.
